# Integrating generative AI and machine learning classifiers for solving heterogenous MCGDM: a case of employee churn prediction

**DOI:** 10.1038/s41598-025-99119-0

**Published:** 2025-05-05

**Authors:** Hagar G. Abu-Faty, Ahmed Kafafy, Mohiy M. Hadhoud, Osama Abdel-Raouf

**Affiliations:** 1 Data Science Department, Faculty of Artificial Intelligence, Minufiya University, Shebeen El-Kom, Egypt; 2 Machine Intelligence Department, Faculty of Artificial Intelligence, Minufiya University, Shebeen El-Kom, Egypt; 3 Information Technology Department, Faculty of Computers and Information, Minufiya University, Shebeen El-Kom, Egypt

**Keywords:** Churn prediction, Generative AI, MCGDM, Machine learning (ML), AHP, TOSIS, GPT, Applied mathematics, Computer science

## Abstract

Employee churn is a critical issue for companies and organizations, as it directly impacts productivity, efficiency, and overall operational success. High turnover rates increase recruitment and training costs, and disrupt workflows, making it a top concern for institutions aiming to maintain stability, growth and continuity. This study presents a methodology to address the employee churn prediction problem in heterogeneous environments by framing it as a Multiple Criteria Group Decision Making (MCGDM) problem. The proposed methodology integrates generative AI, Traditional MCGDM techniques, and machine learning classifiers to handle this problem type. The proposed methodology is structured into four main stages: data collection, generative AI for creating expert profiles, MCGDM for employee ranking, and machine learning for predictive modeling. ChatGPT-4 is used as the generative AI model to simulate expert profiles from diverse fields related to churn prediction. The Analytical Hierarchy Process (AHP) is employed to calculate criteria weights, while the Technique for Order Preference by Similarity to Ideal Solution (TOPSIS) is concerned with alternatives’ ranking and employee’s classification into churn likelihood categories. These rankings data are then used for different nine machine learning classifiers, reducing the computational complexity for future predictions. The results reveal that Neural Networks, Gradient Boosting, and Random Forest outperform other used models in predicting employee churn in terms of accuracy. The proposed methodology offers a scalable, data-driven solution for addressing MCGDM problems, particularly employee churn prediction, by integrating advanced AI techniques with traditional decision-making frameworks.

## Introduction

Group decision making involves multiple individuals coming together to evaluate options and make a collective decision. In such cases, the challenge lies in integrating experts’ varied opinions into a unified decision^1^. When the decision involves assessing alternatives based on several criteria, it evolves into MCGDM problem. In MCGDM, decision-makers must evaluate options using multiple, often conflicting criteria, and each participant may prioritize these criteria differently. This adds another layer of complexity, as the process requires balancing conflicting inputs, assigning weights to the criteria, and combining the group’s preferences into a final decision. MCGDM techniques are essential for addressing such problems, ensuring that diverse viewpoints and criteria are considered in the decision-making process^2^. A wide range of traditional methods is available for solving MCGDM problems. These methods include popular approaches like AHP^3^, TOPSIS^4^, VIKOR^5^, ELECTRE^6^, and PROMETHEE^7^, each offering a unique framework for evaluating alternatives based on multiple criteria. One of the most widely used traditional techniques in MCGDM is the AHP technique^8^. AHP helps decision-makers break down complex problems into a hierarchy of simpler sub-problems. By using pairwise comparisons, it assigns weights to different criteria, which can then be aggregated to evaluate the alternatives. Another popular method is the TOPSIS, which ranks alternatives based on their distance from the positive ideal and the negative-ideal solution. The goal of TOPSIS is to find the alternative closest to the ideal solution and furthest from the least preferred solution^9^. The integration of AHP and TOPSIS provides a structured, transparent, and scalable approach to solving MCDM problems. AHP ensures logical and consistent weight assignment, while TOPSIS offers a fast and efficient ranking process. This hybrid approach is highly effective for making data-driven, interpretable, and objective decisions across various industries^10,11^. While traditional decision-making techniques are effective, they often face challenges in complex, heterogeneous environments. Heterogeneous Group Decision Making (HGDM) involves decision-makers with differing expertise, preferences, opinions, or strategies, each bringing unique perspectives and using varied criteria to assess alternatives^12^. Key features of HGDM include diverse expertise, where decision-makers contribute based on their distinct knowledge and skills, and varied criteria, where each participant may prioritize different factors. Moreover, inconsistent preferences can emerge as participants assign different weights to the same criteria, complicating the aggregation of opinions. These complexities necessitate the use of MCGDM methods to combine diverse inputs and guide the group reach a consensus^13^. To overcome the challenges posed by heterogeneous environments, newer approaches and methods, often leveraging advanced algorithms and artificial intelligence, are being explored to enhance decision-making accuracy and adaptability in complex group decision-making scenarios^14^.

Churn prediction is a critical issue for organizations and companies, as high employee turnover can lead to increased costs, operational disruptions, and reduced productivity. The churn prediction problem involves identifying patterns and factors that influence an employee’s decision to leave, using historical data and analytical techniques^15^. Churn prediction can be effectively addressed as a MCGDM problem, as it requires evaluating employees based on various factors. This process requires input from multiple decision-makers each contributing unique perspectives and priorities which enhance the overall decision quality but it comes with several limitations^16,17^. Gathering a group of experts for decision-making is time-consuming and costly, requiring coordination, compensation, and resources. This makes traditional group decision-making impractical in situations where quick and efficient decisions, like churn prediction, are needed^18^. Many research works have been targeted toward solving Churn prediction problem as a MCDM problem by integrating Machine Learning (ML) techniques to address the limitations of traditional methods as presented in^19–28^ .

The authors in^19^ explores the use of different predictive models for customer churn prediction problems in the telecom industry using six phases and highlights the model’s high accuracy through k-fold cross-validation^20^. proposes a novel framework for employee churn prediction, implemented using the WEKA software. It compares the efficiency and performance of Decision Tree and Logistic Regression models, demonstrating their respective strengths and predictive capabilities in forecasting employee turnover. The use of various machine learning models, such as Naive Bayes, Decision Tree, and Random Forest, is examined to predict employee churn and concludes that Random Forest is the best performing model for employee turnover prediction in^21–23 and 24^​. In^25^ a machine learning model for predicting customer churn in the telecom sector using big data is discussed. It explores feature engineering and social network analysis (SNA) to enhance prediction accuracy, with the XGBoost algorithm achieving the highest performance. A deep learning model based on convolutional neural networks (CNN) for predicting turnover in the workforce, proving its superior accuracy compared to traditional statistical methods​ is explored in^26^. The authors in^27^ introduce a method to predict employee churn by integrating MCDM techniques like CRITIC and MARCOS, and machine learning algorithms, showing that categorizing employees based on their importance improves churn prediction and retention strategies, with the CatBoost algorithm demonstrating the best performance. The authors of^28^ present a novel approach using MCDM techniques, the De-Pareto principle, and machine learning algorithms for categorizing employees and predicting churn.

Generative AI, particularly models like GPT-4, are highly effective tools for creating virtual experts by utilizing their large language model (LLM) capabilities. These AI-driven experts can help address many of the challenges in traditional group decision-making, such as those found in churn prediction problems. By simulating expert decision-making processes, AI can integrate knowledge from various domains and provide a consensus-driven approach without the delays typical of human group dynamics. AI-generated experts are able to process vast amounts of data quickly, utilizing machine learning models, and offer unbiased, data-backed recommendations. Additionally, they can help reduce conflicts stemming from human biases, desires, and backgrounds, as AI systems prioritize objective, data-driven analysis. However, while generative AI enhances decision-making with consistent, scalable outputs, it lacks the intuitive judgment and contextual understanding that human experts provide. As a result, continuous validation is necessary to ensure the AI’s recommendations align with real-world complexities and conditions. The authors of^29^ evaluate GPT-4’s ability to provide medical advice by comparing its responses to those from human medical experts. A thorough evaluation of GPT-4’s translation capabilities compared to human translators is conducted in^30^. The study examines performance across various languages, subject domains, and expertise levels. The authors of^31^ propose a framework integrating GPT-4 with AHP to enhance automated multi-criteria decision support, enabling faster and more consistent decision-making while reducing the need for human intervention. Generative AI, like GPT-4, acts as a virtual expert in employee churn prediction by analyzing complex data sources to provide real-time, data-driven insights and retention strategies, reducing reliance on human experts and enabling faster decision-making.

Predicting churn helps organizations take early steps to keep valuable employees by identifying and addressing the key reasons that might lead them to leave. The challenge is further heightened in heterogeneous environments, as each factor may carry different weights depending on the employee’s position, department, or personal circumstances, making it difficult to arrive at a single, clear decision. This research addresses these complexities by using generative AI, machine learning, and MCGDM techniques. Generative AI emulates experts to generate employees’ evaluation datasets, which are then analyzed using traditional MCGDM methods to rank employees based on churn risk. Machine learning models are subsequently trained to streamline future predictions, reducing computational complexity. This integrated approach helps organizations gain deeper insights into the drivers of turnover and enhances the effectiveness of retention strategies.

The structure of the paper is organized as follows: Sect. 2 provides a detailed explanation of the proposed methodology, Sect. 3 presents the results and discussions related to the proposed methodology, and finally, Sect. 4 concludes the paper and outlines potential directions for future research.

## Methodology

**Fig. 1:**
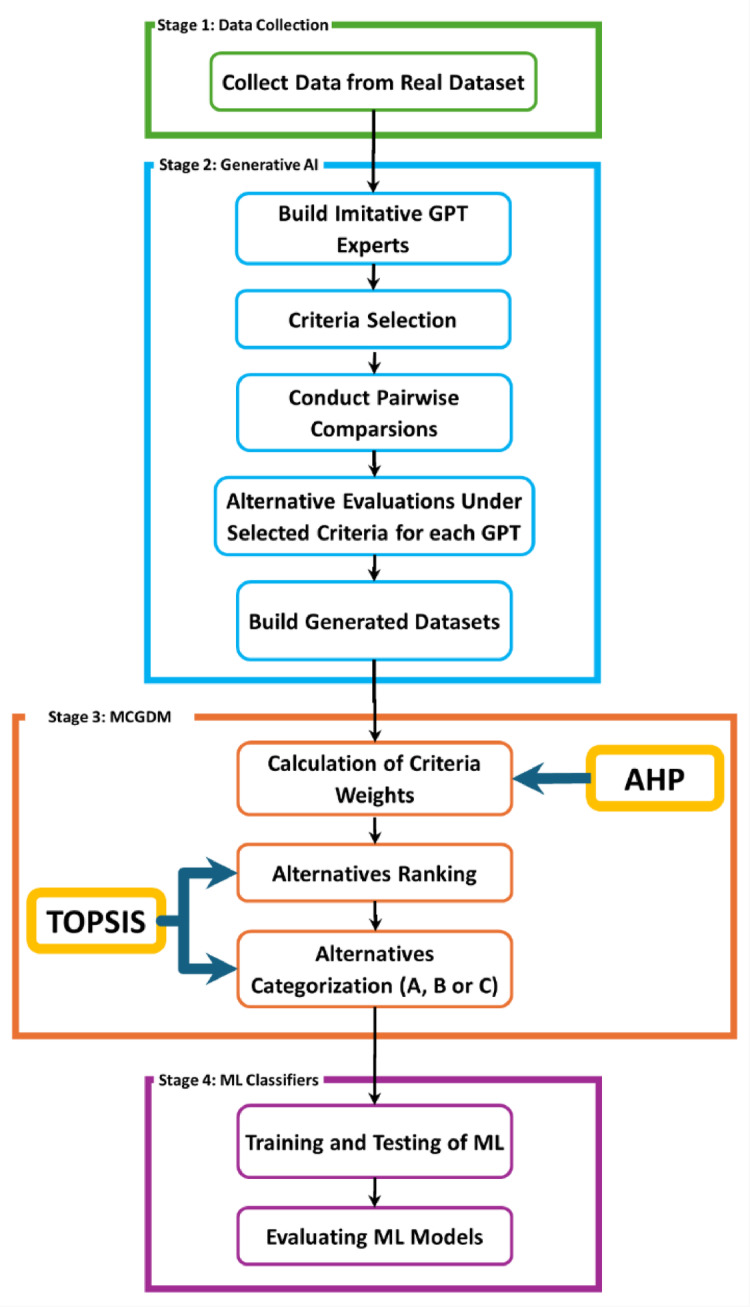
Flow Chart of the Proposed Methodology.

A combination of real and imitative experts is utilized to address the MCGDM problem and rank the alternatives through a series of stages in heterogenous environment. The proposed technique comprises four main stages: data collection, generative AI, Multiple Criteria Group Decision Making (MCGDM), and machine learning for predicting employee churn as illustrated in Fig. 1. In the first stage, Data Collection; relevant employee data is gathered from the actual dataset to be used as a real expert and important features needed for the employee churn prediction process are selected. The second stage, Generative AI utilizes ChatGPT-4’s capabilities to create multiple virtual expert profiles with diverse fields and experiences to simulate real expert decision making process. Key steps in this stage include the selection of relevant criteria for evaluating employee churn, performing pairwise comparisons to determine the relative importance of these criteria, and evaluating employees based on the selected criteria for each virtual expert. These evaluations are then used to generate datasets for subsequent analysis. In the third stage, MCGDM Techniques are employed to solve the churn prediction problem. AHP is used to determine the weight of each selected criterion, followed by TOPSIS to rank alternatives based on the weighted criteria. Employees are then classified into categories (A, B, or C) reflecting their likelihood of churn. The output obtained from this stage is utilized to gather data for training and testing machine learning algorithms in the subsequent stage. Finally, Stage 4 focuses on ML Classifiers, where various machine learning models are trained and tested on the datasets to function as experts for predicting employee turnover. The performance of these models is thoroughly evaluated to ensure prediction accuracy.

### Data collection and preprocessing

The proposal is based on the IBM Watson dataset, published in 2017 by the Smarter Workforce Institute at IBM, which contains approximately 19,479 employee records and 31 data attributes with no missing values. This structured, tabular dataset is specifically designed for employee churn prediction, helping to determine whether an employee is likely to leave the company based on various factors. The dataset includes a range of work-related attributes, such as job performance, role within the company, job involvement, monthly income, overtime status, and years at the company, as well as personal factors, including age, education level, marital status, and work-life balance. Additionally, it incorporates employee feedback on job satisfaction, workplace environment, and relationships within the company, making it a comprehensive resource for churn analysis. The dataset’s features are categorized into numerical, ordinal, and categorical types, each playing a crucial role in churn prediction. Numerical features include Age, Number of Companies Worked, Hourly Rate, Monthly Income and other attributes. Ordinal features include Education Level (ranging from 1 to 5), Job Satisfaction (ranging from 1 to 4) and Work-Life Balance (from 1 to 4) and etc. Categorical features can include Gender (Male/ Female), MaritalStatus (Single, Married, Divorced), and Department (HR, Sales, or R&D) and so on. Given its depth and reliability, the dataset is treated as a real expert in the proposed technique, serving as a foundational source for model training and evaluation.

Data preprocessing is a vital step in preparing the dataset to be clean, structured, and well-prepared for machine learning models. The process begins with checking for missing values to confirm that the dataset is complete. Next, duplicate records are detected and removed to eliminate bias and redundancy, improving the model’s learning efficiency. Ordinal features are then validated and mapped into appropriate numerical values for accurate representation. Following this, all data is standardized using the Saaty scale, ensuring consistency across features, as machine learning models perform optimally when numerical attributes are on the same scale. This transformation enhances the dataset’s suitability for ranking alternatives in the MCGDM problem. Finally, the dataset is split into two parts (80:20 ratio), with 80% allocated for training and 20% for testing machine learning classifiers, facilitating robust model evaluation and improving predictive performance.

### Feature selection

The proposed technique employs a multi-stage feature selection approach that incorporates the capabilities of Generative AI (ChatGPT-4) and MCDM techniques to identify the most significant features for employee churn prediction. Initially, ChatGPT-4 was utilized to generate multiple virtual experts including HR specialists and data analysts, who contributed domain-specific insights to analyze the IBM Waston dataset and identify the key attributes influencing employee churn. Each expert contributed insights based on their domain knowledge ensuring that feature selection was data-driven rather than assumption-based, enhancing objectivity and eliminating human bias. By analyzing the IBM Watson dataset, the AI model identified 15 key features with the highest impact on employee churn, including Job Satisfaction, Monthly Income, OverTime, Years at Company, Work-Life Balance, and Environment Satisfaction. As the employee evaluation is a heterogeneous MCGDM problem, different experts with varied expertise and backgrounds employ distinct evaluation criteria and methods to assess alternatives. Each expert determines the most suitable criteria based on their experience, ensuring a comprehensive and balanced evaluation. Once the key features were selected for each expert, AHP method was applied to prioritize these features by performing a pairwise comparison, assigning relative importance scores, and ensuring logical consistency through Consistency Ratio (CR) calculations. The primary steps of the feature selection process are illustrated in Fig. 1, while Sect. 2.3 provides a detailed explanation of the feature selection methodology based on the Generative AI, and Sect. 2.4 elaborates on the criteria weighting method.

### Generative AI

In this research, a newly released feature from OpenAI, which enables the creation of custom GPT models, is utilized. This feature allows the model to be tailored with specific instructions, foundational documentation to be provided, and defined limitations to be set, optimizing the model for particular tasks.

Generative AI represented by GPTs, is employed to generate a set of imitative experts based on the basic information about employees in the provided dataset. On the other hand, the insights and evaluations of the real expert are derived directly from the data within the dataset.

Firstly, a custom ChatGPT model named “Expert Guide” was created to act as the primary decision-maker for the key steps in the AHP model development. This includes identifying the optimal number of virtual experts to be generated and their areas of expertise for solving the problem. Also, Identifying the most relevant criteria from the dataset for the real expert. It can also be used to conduct pairwise comparisons needed by AHP to determine the weights of criteria.

“Expert Guide” GPT was consulted to determine the optimal number of experts required for solving the churn prediction problem and responded with a recommendation of seven experts from various relevant domains. This number is suggested because it balances the need for diverse perspectives with the practicality of managing inputs and synthesizing the results.

The recommended experts and their respective fields are summarized as follows:

Expert 1: Behavioral Data Scientist.

Expert 2: Workforce Analytics Specialist.

Expert 3: Senior HR Consultant.

Expert 4: Labor Economist.

Expert 5: Employee Engagement Strategist.

Expert 6: Employee Relations Specialist.

Expert 7: Data Scientist and Machine Learning Expert.

By incorporating expert knowledge across these areas, a well-rounded and insightful AHP model will be built for solving employee churn prediction problem.

The “Expert Guide” recommendation emphasizes the importance of the Behavioral Data Scientist and Senior HR Consultant, as their expertise is crucial for understanding the reasons behind employee turnover and designing effective interventions. Significant importance is also given to the Workforce Analytics Specialist and Employee Engagement Strategist, who offer vital data-driven insights and strategies that can directly impact churn outcomes. Meanwhile, the Labor Economist, Employee Relations Specialist, and Data Scientist play valuable and supportive roles. Their contributions, while essential, are more context-specific, helping to fine-tune the solutions developed by the primary experts.

After considering the number of virtual experts needed to address the problem, custom GPT personas were created based on the virtual profiles provided by the “Expert Guide.” After determining the number of virtual experts. Each expert focuses on a unique area related to churn prediction and employee retention. Expert 1 specializes in data science to analyze employee behavior patterns, identifying early predictors of churn and proactively addressing potential risk factors. Expert 2 develops and applies performance and engagement metrics specifically for churn prediction, offering actionable insights to reduce turnover. Expert 3 advises on HR practices that mitigate turnover risk, using churn analysis insights to enhance retention strategies. Expert 4 examines economic trends and labor market dynamics affecting turnover rates, helping to study churn within broader labor market conditions. Expert 5 identifies and addresses factors related to engagement and job satisfaction that directly impact employee retention. Expert 6 intervenes in potential churn cases by managing the relationship between employees and the organization, handling conflicts, and ensuring workplace regularity through effective communication and policies. Expert 7 utilizes machine learning and predictive models to analyze historical data, identifying patterns and factors that signal turnover risk. Each expert contributes a distinct set of insights and approaches to build a comprehensive strategy for predicting and mitigating employee churn. As a result of generating the seven GPTs, now the problem has eight experts, one is a real expert based on the information given in the dataset and seven imitative by ChatGPT capabilities.

After that, The “Expert Guide” is tasked with identifying the most effective criteria considering information about all employees in the dataset to be utilized by the real expert. A set of criteria is selected that has the greatest impact on the employee turnover process according to the dataset. The selected criteria are EmpEnvironmentSatisfaction, EmpJobInvolvement, EmpJobSatisfaction, RelationshipSatisfaction, and EmpWorkLifeBalance. For simplicity, these criteria are listed as Work Environment, Engagement Levels, Job Satisfaction, Peer Interactions, and Work-life Balance.

**Fig. 2:**
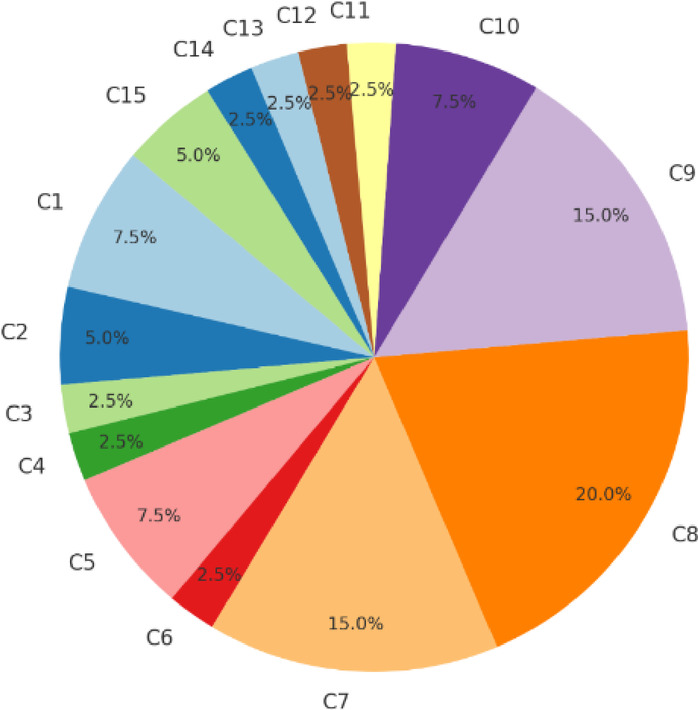
Criteria Voting Percentages.

Once the seven experts are generated, each of them is asked to propose a number of evaluation criteria for employee churn prediction according to their specific field of expertise. Our imitative experts each suggested an extensive list of 5 primary criteria, ultimately resulting in a total of 15 unique top-level criteria without duplication. The suggested criteria for all experts are summarized in Table 1. The distribution of decision-makers’ evaluations across 15 criteria (C1 to C15) is shown in Fig. 2. Each slice of the pie chart represents the percentage of votes attributed to each criterion. C7, C8, and C9 have the highest percentage of votes, indicating that these criteria are the most significant in the decision-making process. On the other hand, C3, C4, C6, C11, C12, C13, and C14 have each received 2.5% of the total votes, making them less significant compared to others.

**Table 1:** Criteria selected for each expert.

Criteria		Real Expert	Expert 1	Expert 2	Expert 3	Expert 4	Expert 5	Expert 6	Expert 7
C1	Engagement Levels	✓	✓	✓					
C2	Peer Interactions	✓	✓						
C3	Performance Metrics		✓						
C4	Sentiment Trends		✓						
C5	Compensation Competitiveness			✓	✓	✓			
C6	Performance Consistency			✓					
C7	Career Progression			✓	✓	✓	✓	✓	✓
C8	Work-life Balance	✓	✓	✓	✓	✓	✓	✓	✓
C9	Job Satisfaction	✓			✓	✓	✓	✓	✓
C10	Managerial Support				✓			✓	✓
C11	Compensation Structure								✓
C12	Management Practices					✓			
C13	Recognition and Rewards						✓		
C14	Feedback Systems Quality						✓		
C15	Work Environment	✓						✓	

Following this, each expert is asked to conduct pairwise comparisons of their selected criteria to calculate the criteria weights using the AHP technique.

As a final step for generative AI, the experts are asked to evaluate the employees in the provided dataset using the Saaty scale, ranging from 1 to 9. Each expert assesses the criteria as if they were human, drawing on their background and experience to provide a comprehensive evaluation.

### MCGDM techniques

AHP and TOPSIS are two widely used techniques for solving MCGDM problems. In this stage, AHP is applied to calculate the criteria weights for each decision maker. These weights are then passed to TOPSIS, which ranks the employees in the dataset based on their likelihood of turnover.

The integration of AHP and TOPSIS provides a structured, transparent, and efficient framework for ranking employees based on their likelihood of churn. AHP determines the importance of different churn factors through a hierarchical structure and pairwise comparisons, ensuring that qualitative factors are systematically evaluated. Additionally, the Consistency Ratio test enhances the reliability of feature selection by maintaining logical consistency in expert judgments. Once AHP assigns criteria weights, TOPSIS ranks employees based on their proximity to an ideal retention scenario and distance from a worst-case churn scenario, offering clear, data-driven predictions using Euclidean distance calculations. TOPSIS considers all criteria simultaneously, providing a balanced ranking and ensuring computational efficiency, making it ideal for large-scale HR analytics.

By combining AHP’s structured weight assignment with TOPSIS’s efficient ranking system, this approach merges subjective decision-making with objective ranking, enabling interpretable and data-driven employee retention strategies. Furthermore, integrating Generative AI (GAI) with AHP-TOPSIS automates expert evaluations, enhances ranking accuracy, and enables real-time churn predictions. This combination minimizes human bias, improves decision-making, and ensures a scalable, data-driven workforce management strategy, allowing organizations to proactively identify churn risks and implement effective retention strategies.

#### AHP technique

**Fig. 3:**
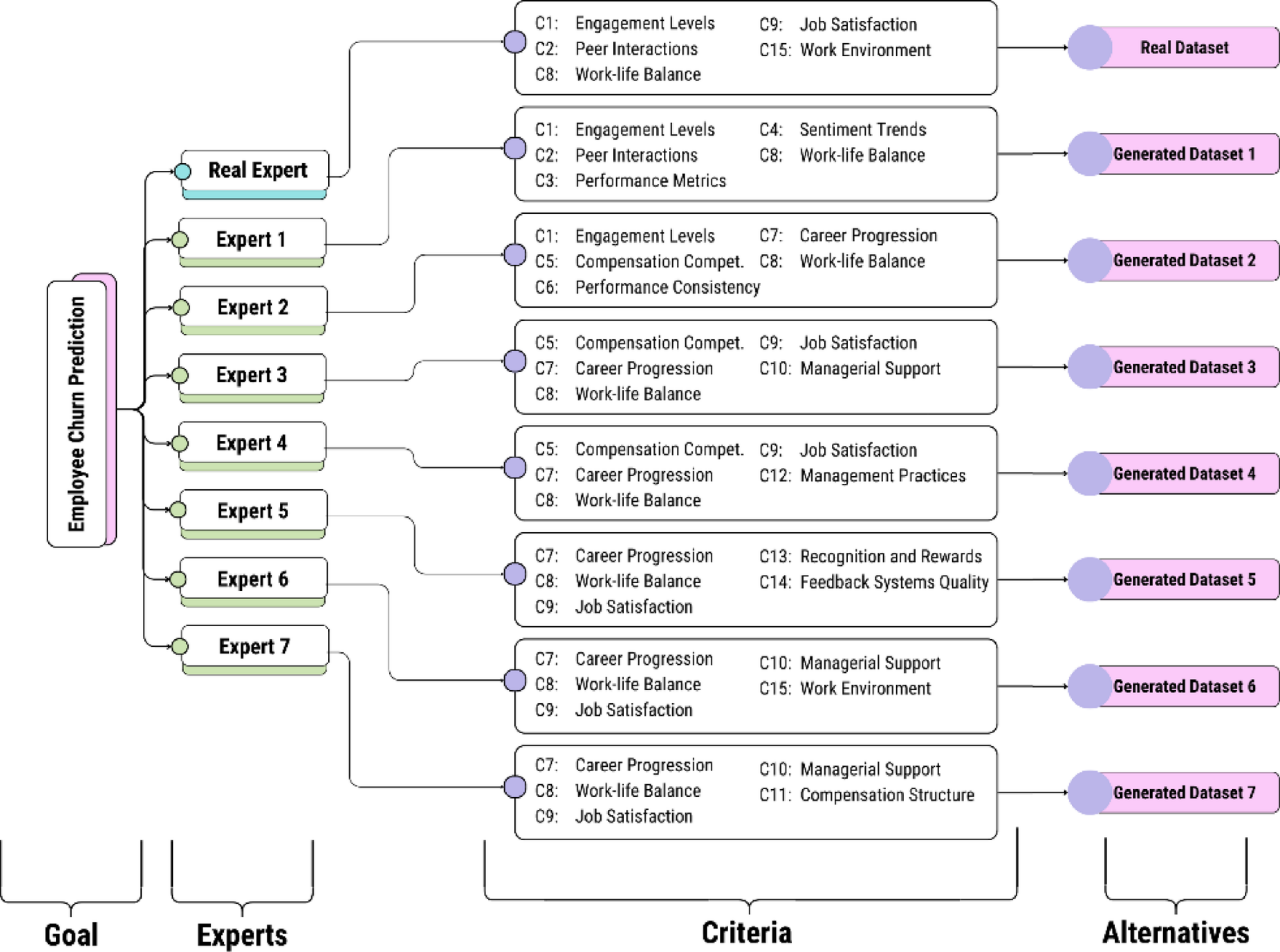
The Complete AHP Hierarchy Tree.

After generating decision matrices for each expert using generative AI, the problem now consists of eight distinct decision matrices; seven obtained from the virtual experts and one from the original dataset, which serves as the real expert. The complete AHP hierarchy is displayed in Fig. 3. Each expert GPT is tasked with conducting pairwise comparisons of their selected criteria to calculate the criteria weights using the AHP technique. Additionally, the “Expert Guide” is asked to perform a pairwise comparison of the criteria selected from the original dataset, which will be assigned to the real expert. The AHP technique is applied to calculate the criteria weights based on the pairwise comparisons provided by the experts. Considering the overlaps between the criteria, the final weight for each criterion will be determined using Eqs. (1) and (2).

Consider a multi-criteria group decision-making problem with $\:m\:$alternatives, $\:n\:$criteria, and $\:p\:$decision makers. Let $\:DM=\left\{{DM}_{1},{DM}_{2},{\dots\:,\:DM}_{p}\right\}$ be the set of $\:p$ decision makers. $\:A=\left\{{A}_{1},{A}_{2},{\dots\:,A}_{m}\right\}$ be a set of $\:m$ alternatives and $\:C=\left\{{C}_{1},{C}_{2},{\dots\:,C}_{n}\right\}$ be the set of $\:n\:$criteria.$\:\:{W}_{ij}=\left\{{W}_{1},{W}_{2},{\dots\:,W}_{n}\right\}\:$be the set of criteria weights assigned by decision maker $\:i$ to criterion $\:j$

1. Calculate the weighted sum for each criterion by Eq. (1)

1 $${S_j}=\sum\limits_{{i=1}}^{p} {{W_{ij}} * {N_{ij}}}$$

Where *S*_*j*_ is the weighted sum for criterion *C*_*j*_. *W*_*ij*_ is the weight assigned by decision maker *i* to criterion *j*. *N*_*ij*_ is the number of decision makers evaluating criterion j.

2. Calculate the final weight for each criterion by Eq. (2)

2 $${W_j}=\frac{{{S_j}}}{{\sum\nolimits_{{j=1}}^{n} {{S_j}} }}$$

Where *W*_*j*_ is the final weight for criterion *C*_*j*_.

**Fig. 4:**
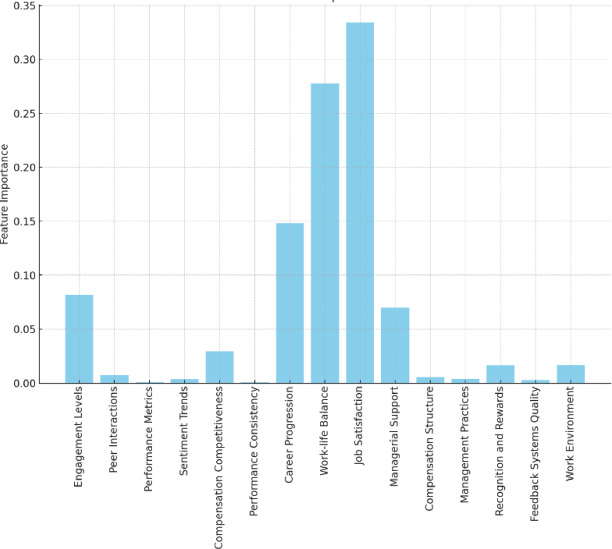
Criteria Importance.

The weights determined by each expert along with final criteria weights are presented in Table 2. The criteria weights represent the significance of the features used in conjunction with the machine learning models. The importance of these criteria or features is displayed in Fig. 4.

**Table 2:** Criteria weights.

Criteria		Real Expert	Expert 1	Expert 2	Expert 3	Expert 4	Expert 5	Expert 6	Expert 7	W_j_
C1	Engagement Levels	0.2571	0.4578	0.503						0.08187
C2	Peer Interactions	0.0858	0.083							0.007565
C3	Performance Metrics		0.0413							0.000925
C4	Sentiment Trends		0.1621							0.003632
C5	Compensation Competitiveness			0.26	0.109	0.071				0.029578
C6	Performance Consistency			0.035						0.000784
C7	Career Progression			0.134	0.167	0.11	0.1332	0.471	0.088	0.148318
C8	Work-life Balance	0.3145	0.2559	0.068	0.233	0.183	0.2365	0.1532	0.106	0.277869
C9	Job Satisfaction	0.2271			0.423	0.46	0.4309	0.4558	0.489	0.3342
C10	Managerial Support				0.067			0.901	0.074	0.070045
C11	Compensation Structure								0.242	0.005423
C12	Management Practices					0.177				0.003966
C13	Recognition and Rewards						0.7347			0.016463
C14	Feedback Systems Quality						0.1259			0.002821
C15	Work Environment	0.1153						0.2538		0.016541

#### TOPSIS

The problem is now formulated as a MCGDM problem involving 8 experts, 15 criteria, and a set of different alternatives or employees. The weights for all criteria have been determined using the AHP technique. The primary role of TOPSIS is to rank employees in order to identify those most likely to leave the organization. TOPSIS seeks to find the alternative that is as close as possible to the best ideal solution while being as far as possible from the worst ideal solution. This makes it a powerful and intuitive method for solving MCGDM problems. The main steps of TOPSIS are summarized as follows:

1. Construction of the decision matrices based on decision makers’ assessments.

Each decision maker (DM) creates their own decision matrix based on their expertise and perspective, using the generative AI framework as explained earlier. Each matrix includes the evaluation of all alternatives (employees) against the selected criteria, with values representing the performance or suitability of each alternative relative to each criterion.

2. Construction of the aggregated decision matrix Using the Geometric Mean Operator:

The aggregated decision matrix $\:{D=[d}_{ij}]\:$is calculated using Eq. (3)

3 $$D=\left[ {{d_{ij}}} \right]={\left( {\prod\limits_{{k=1}}^{{{n_{ij}}}} {{X_{ijk}}} } \right)^{{\raise0.7ex\hbox{$1$} \!\mathord{\left/ {\vphantom {1 {{n_{ij}}}}}\right.\kern-0pt}\!\lower0.7ex\hbox{${{n_{ij}}}$}}}}$$

Where [*d*_*ij*_] is the aggregated value for alternative *i* under criterion *j*. *X*_*ijk*_ is the evaluation provided by expert *k* for alternative *i* under criterion *j*. *n*_*ij*_ is the number of experts who evaluated alternative *i* under criterion *j*.

3. Normalization of the Aggregated Decision Matrix:

The normalized matrix $\:{{[d}_{ij}}^{N}]$ is computed as Eq. (4)

4 $$\left[ {{d_{ij}}^{N}} \right]=\frac{{{d_{ij}}}}{{\sqrt {\sum\nolimits_{{i=1}}^{m} {{d_{ij}}^{2}} } }}$$

4. Construction of the weighted normalized decision matrix.

The weighted normalized decision matrix $\:{{[d}_{ij}}^{*}]\:$is calculated as in Eq. (5)

5 $$\left[ {{d_{ij}}^{*}} \right]=\left[ {{d_{ij}}^{N}} \right] * {w_j}$$

5. Identifying of the positive-ideal (PIS) and the negative-ideal (NIS) solutions using Eq. (6)

PIS ($\:{\text{d}}^{+}$) is the best possible solution, where NIS ($\:{\text{d}}^{-}$) is the worst possible solution. They can be obtained by:

6 $$\begin{gathered} {d^+}=\hbox{max} \left( {{d_{ij}}^{*}} \right){\text{ for benefit criteria, }}\hbox{min} \left( {{d_{ij}}^{*}} \right){\text{ for cost criteria}} \hfill \\ {d^ - }=\hbox{min} \left( {{d_{ij}}^{*}} \right){\text{ for benefit criteria, }}\hbox{max} \left( {{d_{ij}}^{*}} \right){\text{ for cost criteria}} \hfill \\ \end{gathered}$$

6. Calculation of the distance measure:

For each alternative, the distance measure to the PIS $\:\left({S}_{i}^{+}\right)$ is measured by Eq. (7), and to the NIS $\:\left({S}_{i}^{-}\right)$ by Eq. (8) based on the Euclidean distance measure.

7 $${S_i}^{+}=\sqrt {\sum\limits_{{j=1}}^{n} {{{\left( {{d_{ij}}^{*} - {d^+}} \right)}^2}} }$$

8 $${S_i}^{ - }=\sqrt {\sum\limits_{{j=1}}^{n} {{{\left( {{d_{ij}}^{*} - {d^ - }} \right)}^2}} }$$

7. Calculation of the relative closeness coefficient to the ideal solution by Eq. (9)

9 $$C{C_i}=\frac{{{S_i}^{ - }}}{{{S_i}^{+}+{S_i}^{ - }}}{\text{ where, }}0 \leqslant C{C_i} \leqslant 1$$

8. Ranking the alternatives.

Alternatives are ranked based on their closeness coefficient value, with employees ranked higher being more likely to leave the organization.

**Fig. 5:**
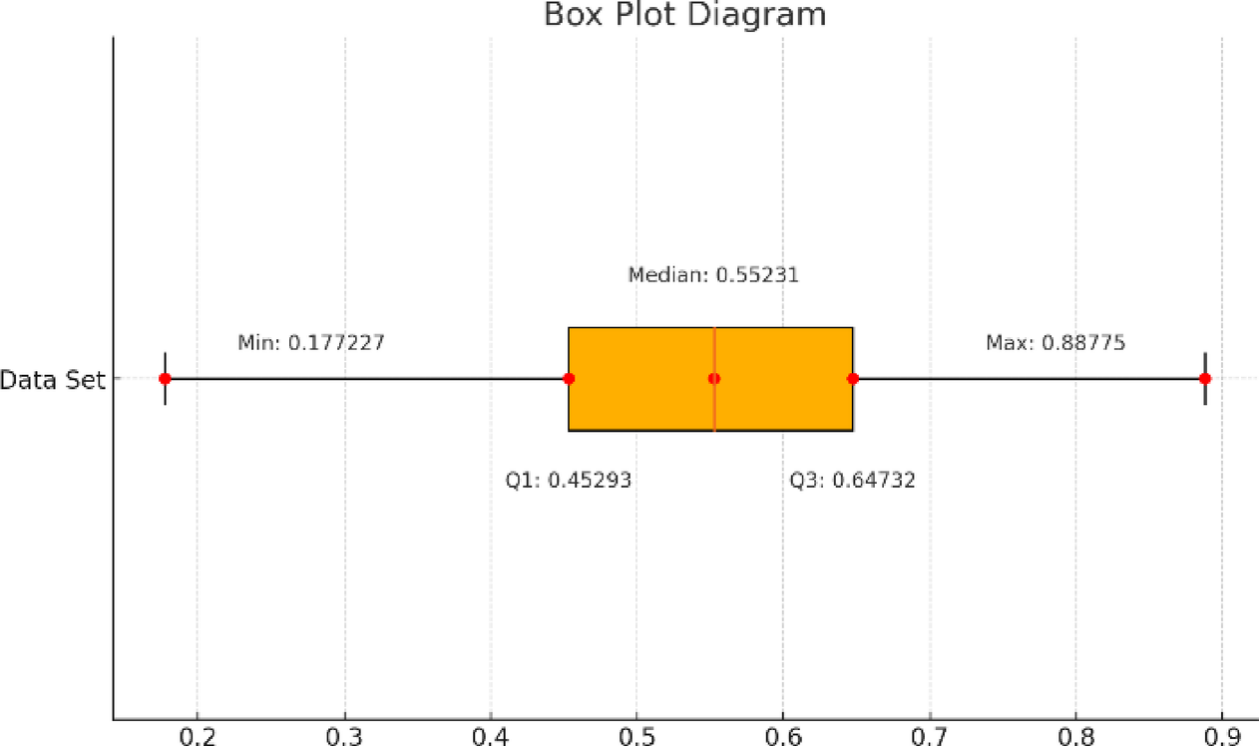
Box Plot of Ranked Employees.

After ranking the employees, they are classified into three categories based on data derived from a box plot shown in Fig. 5. The first category includes those with the greatest likelihood of turnover. Table 3 presents the classification of employee categories. The first category, Class A, with the highest rankings, identified by a closeness coefficient greater than the third quartile value (75th Percentile) (Q3 = 0.64732) obtained from the box plot. This group represents employees at high risk of turnover based on churn prediction. Class C represents the lowest risk group for leaving the organization, with closeness coefficients below the first quartile (25th Percentile) (Q1 = 0.45300). Lastly, Class B, comprises those at moderate risk, with closeness coefficients between the first and third quartiles (Q1 and Q3).

**Table 3:** Employee categories.

Category A	CC > Q3	The upper 25% of the employees
Category B	CC > Q1 & CC < Q3	The middle 50% of the employees
Category C	CC < Q1	The lower 25% of the employees

### Machine learning classification

After obtaining the employee rankings and classification categories for churn prediction using TOPSIS, the next step is to apply various machine learning algorithms to the datasets to validate the effectiveness of using ML for solving this problem.

ML classification is based on four key concepts: classes, features, training, and testing. The classes represent the target categories the model aims to predict. For the churn prediction problem, there are three classes: A, B, and C. Features are the criteria used to make the predictions about the target. In the case of study, 15 different criteria are considered for prediction. The datasets provided by decision makers are used in training process where the classification algorithm learns from labeled data by identifying patterns and relationships between the features and their corresponding classes. After training, the model is evaluated on a testing dataset to assess its accuracy in predicting the correct class.

In this approach, nine different types of supervised learning classifiers are used to categorize employees into one of three categories, similar to TOPSIS method. The training process relies on evaluations obtained from both the real dataset and the generated datasets from imitative experts. The primary goal for applying ML classifiers to the churn prediction problem is to accurately predict potential turnover and deliver actionable insights to help minimize losses.

#### ML classifiers’ parameters

Nine different classifiers are applied to the churn prediction problem to categorize employees into three groups, identifying those most likely to leave the organization.

These classifiers are AdaBoost, Gradient Boosting (CatBoost), and Random Forest, Logistic Regression, KNN, and Neural Network, SVM, Decision Tree, and Naïve Bayes.

In the proposed methodology, the working parameters of ML classifiers are stated as follows: AdaBoost utilizes 60 estimators using decision trees as the base estimator, the Samme. R algorithm for classification, and a linear loss function for regression tasks. Gradient Boosting (CatBoost) employs 170 trees, a learning rate of 0.3, a maximum tree depth of 15, with replicable training and a regularization strength of 3. Random Forest runs with 150 trees, without limits on features or tree depth. It stops splitting nodes with 5 instances, but no replicable training is supported. Logistic Regression takes a simple approach with Ridge (L2) regularization, a C value of 1, and no class weights, ensuring balanced predictions. KNN keeps things basic with 5 neighbors, utilizing the Euclidean distance metric, and uniform weighting for classification. The Neural Network model is configured with two hidden layers of 50 neurons each. It uses the tanh activation function with the Adam solver, allowing up to 500 iterations with some replicability in training. For SVM, a radial basis function (RBF) kernel with a C-value of 1.0 and a tight numerical tolerance of 0.001is used. It’s set for 100 iterations, ensures a balance between precision and computational efficiency. The Decision Tree is pruned to keep at least 2 instances in leaves and 5 in internal nodes, with a maximum depth of 100, stopping splits when 95% of instances in a node belong to the same class. All these models have been fine-tuned to maximize precision, efficiency, and accuracy, making them highly effective for churn prediction problems.

## Results and discussion

The Pareto Principle, also known as the 80:20 Rule, suggests that approximately 80% of the effects come from 20% of the causes. In line with this concept, the dataset was initially split into 80:20 ratio, with 80% used for training the classifiers and 20% reserved for testing the ML models. To further evaluate the performance of the models on training data, a 10-fold cross-validation was performed. Table 4 presents the performance measures of the ML models after splitting the dataset into an 80:20 ratio and categorizing the employees into three distinct groups.

**Table 4:** ML performance measures (80:20 Split).

Classifier	Classes	Precision	Recall	F1-score	Accuracy	Accuracy %
Tree	All Classes	0.876	0.875	0.875	0.875	87.5%
	Class A	0.876	0.859	0.867	0.875	
	Class B	0.872	0.880	0.876	0.875	
	Class C	0.883	0.882	0.882	0.875	
SVM	All Classes	0.830	0.823	0.824	0.823	82.3%
	Class A	0.730	0.887	0.801	0.823	
	Class B	0.858	0.809	0.833	0.823	
	Class C	0.874	0.786	0.828	0.823	
Random Forest	All Classes	0.908	0.908	0.908	0.908	90.8%
	Class A	0.929	0.899	0.914	0.908	
	Class B	0.895	0.923	0.909	0.908	
	Class C	0.913	0.884	0.898	0.908	
Neural Network	All Classes	0.953	0.953	0.953	0.953	95.3%
	Class A	0.950	0.952	0.951	0.953	
	Class B	0.951	0.954	0.953	0.953	
	Class C	0.958	0.951	0.954	0.953	
Naïve Bayes	All Classes	0.680	0.649	0.634	0.649	64.9%
	Class A	0.612	0.897	0.728	0.649	
	Class B	0.754	0.446	0.561	0.649	
	Class C	0.598	0.807	0.687	0.649	
Logistic Regression	All Classes	0.857	0.856	0.856	0.856	85.6%
	Class A	0.886	0.847	0.866	0.856	
	Class B	0.838	0.882	0.860	0.856	
	Class C	0.866	0.813	0.839	0.856	
KNN	All Classes	0.823	0.823	0.823	0.823	82.3%
	Class A	0.820	0.797	0.808	0.823	
	Class B	0.811	0.844	0.827	0.823	
	Class C	0.852	0.806	0.828	0.823	
Gradient Boosting	All Classes	0.928	0.928	0.928	0.928	92.8%
	Class A	0.946	0.900	0.923	0.928	
	Class B	0.915	0.943	0.929	0.928	
	Class C	0.937	0.925	0.931	0.928	
AdaBoost	All Classes	0.861	0.861	0.861	0.861	86.1%
	Class A	0.864	0.854	0.859	0.861	
	Class B	0.860	0.861	0.861	0.861	
	Class C	0.859	0.867	0.863	0.861	

Based on the results from the 80:20 split, Neural Networks and Gradient Boosting consistently demonstrate the best performance across all classes, with high F1-scores, precision, recall, and accuracy. Random Forest also performs exceptionally well, ranking just slightly behind Gradient Boosting and Neural Networks. Naïve Bayes, however, shows the weakest performance across all metrics, particularly struggling with precision, making it less suitable for this churn prediction problem. SVM and KNN offer moderate performance, with SVM facing challenges in Class A, while KNN provides more consistent results across all classes. Logistic Regression and AdaBoost are both provide a solid balanced performance in all categories.

In terms of accuracy:

- Neural Networks achieved the highest accuracy at 95.3%,
- Gradient Boosting followed with 92.8%,
- Random Forest achieved 90.8% accuracy.

**Fig. 6:**
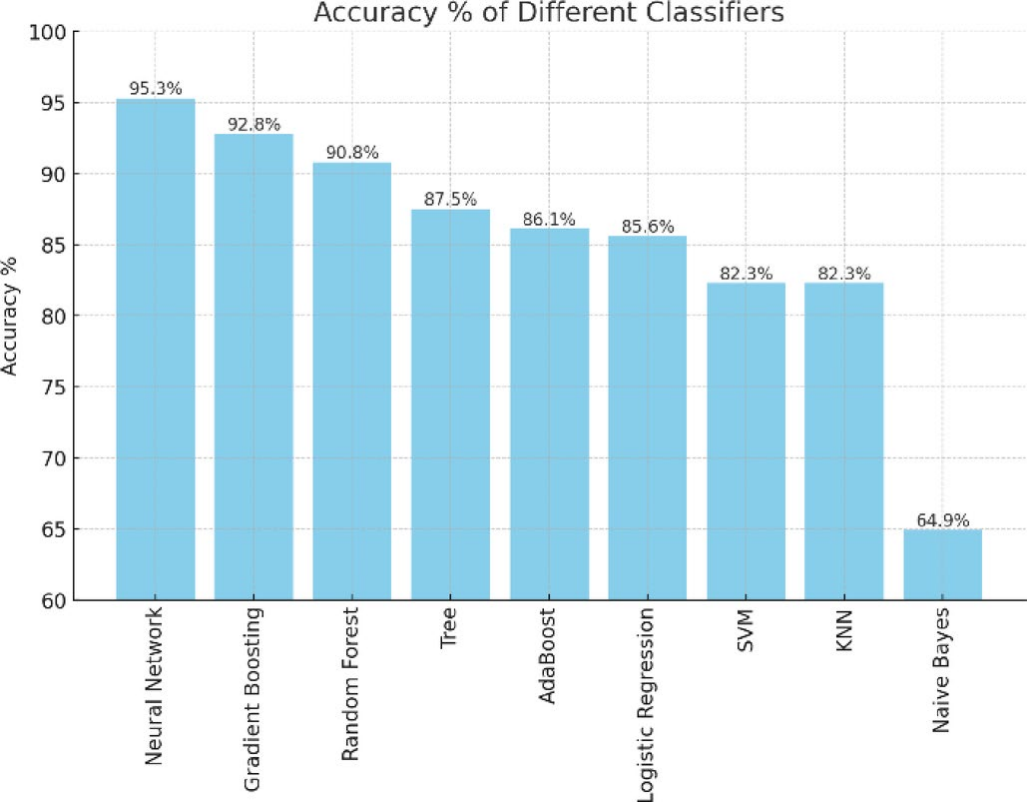
Accuracy Percentages for All Classifiers.

**Fig. 7:**
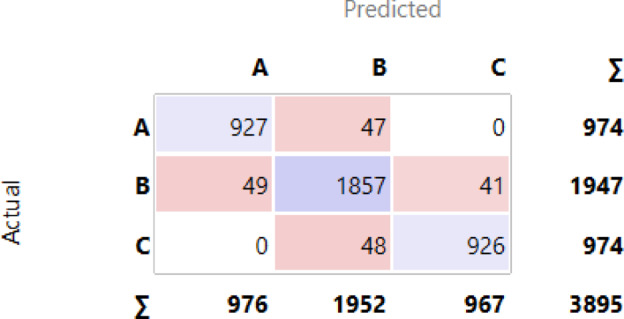
Confusion Matrix for Neural Network.

**Fig. 8:**
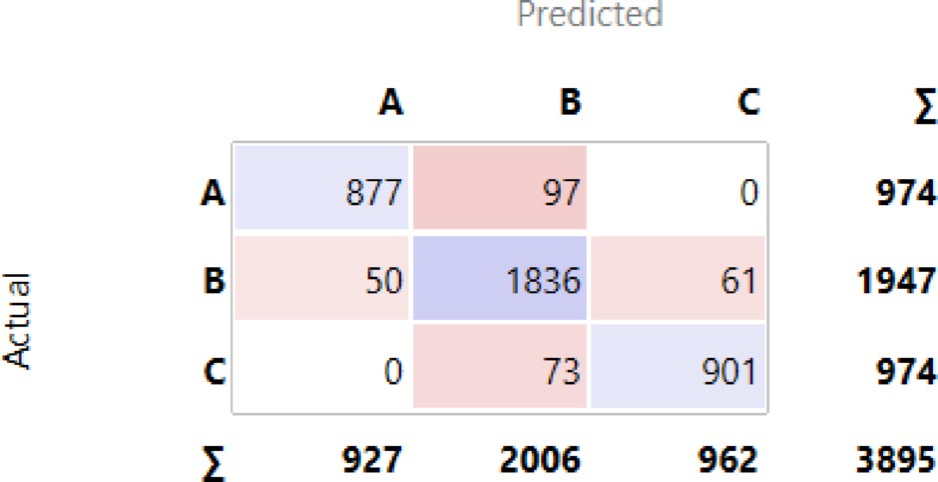
Confusion Matrix for Gradient Boosting.

**Fig. 9:**
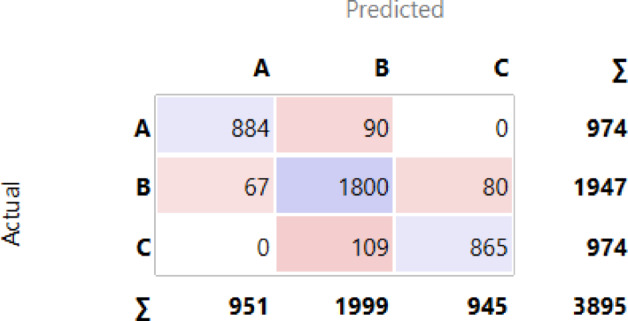
Confusion Matrix for Random Forest.

These three models stand out as the top performers, with the choice of classifier depending on the specific needs of the churn prediction problem. The percentage of accuracy for all classifiers, ordered descending, is displayed in Fig. 6. The confusion matrices for the three top classifiers are illustrated in Figs. 7, 8 and 9, respectively.

Additionally, the evaluation metrics of the ML models were assessed using 10-fold cross-validation to ensure robustness and reliability. The results of this evaluation, including key performance indicators such as accuracy, precision, recall, and F1-score, are comprehensively illustrated in Table 5.

**Table 5:** ML performance measures (10-fold cross-validation).

Classifier	Classes	Precision	Recall	F1-score	Accuracy	Accuracy %
Tree	All Classes	0.865	0.865	0.865	0.865	86.5%
	Class A	0.855	0.862	0.859	0.865	
	Class B	0.867	0.862	0.865	0.865	
	Class C	0.871	0.873	0.872	0.865	
SVM	All Classes	0.821	0.819	0.820	0.819	81.9%
	Class A	0.754	0.817	0.784	0.819	
	Class B	0.836	0.830	0.833	0.819	
	Class C	0.859	0.801	0.829	0.819	
Random Forest	All Classes	0.903	0.902	0.902	0.902	90.2%
	Class A	0.911	0.903	0.907	0.902	
	Class B	0.886	0.924	0.905	0.902	
	Class C	0.931	0.858	0.893	0.902	
Neural Network	All Classes	0.953	0.953	0.953	0.953	95.3%
	Class A	0.946	0.967	0.956	0.953	
	Class B	0.956	0.950	0.953	0.953	
	Class C	0.954	0.945	0.949	0.953	
Naïve Bayes	All Classes	0.672	0.637	0.618	0.637	63.7%
	Class A	0.574	0.887	0.697	0.637	
	Class B	0.749	0.417	0.536	0.637	
	Class C	0.615	0.825	0.705	0.637	
Logistic Regression	All Classes	0.865	0.865	0.865	0.865	86.5%
	Class A	0.870	0.879	0.874	0.865	
	Class B	0.858	0.875	0.866	0.865	
	Class C	0.875	0.832	0.853	0.865	
KNN	All Classes	0.820	0.820	0.820	0.820	82.0%
	Class A	0.804	0.809	0.807	0.820	
	Class B	0.819	0.824	0.822	0.820	
	Class C	0.839	0.823	0.831	0.820	
Gradient Boosting	All Classes	0.923	0.922	0.922	0.922	92.2%
	Class A	0.930	0.924	0.927	0.922	
	Class B	0.909	0.938	0.924	0.922	
	Class C	0.943	0.889	0.915	0.922	
AdaBoost	All Classes	0.854	0.854	0.854	0.854	85.4%
	Class A	0.836	0.867	0.851	0.854	
	Class B	0.860	0.845	0.852	0.854	
	Class C	0.860	0.858	0.859	0.854	

**Fig. 10:**
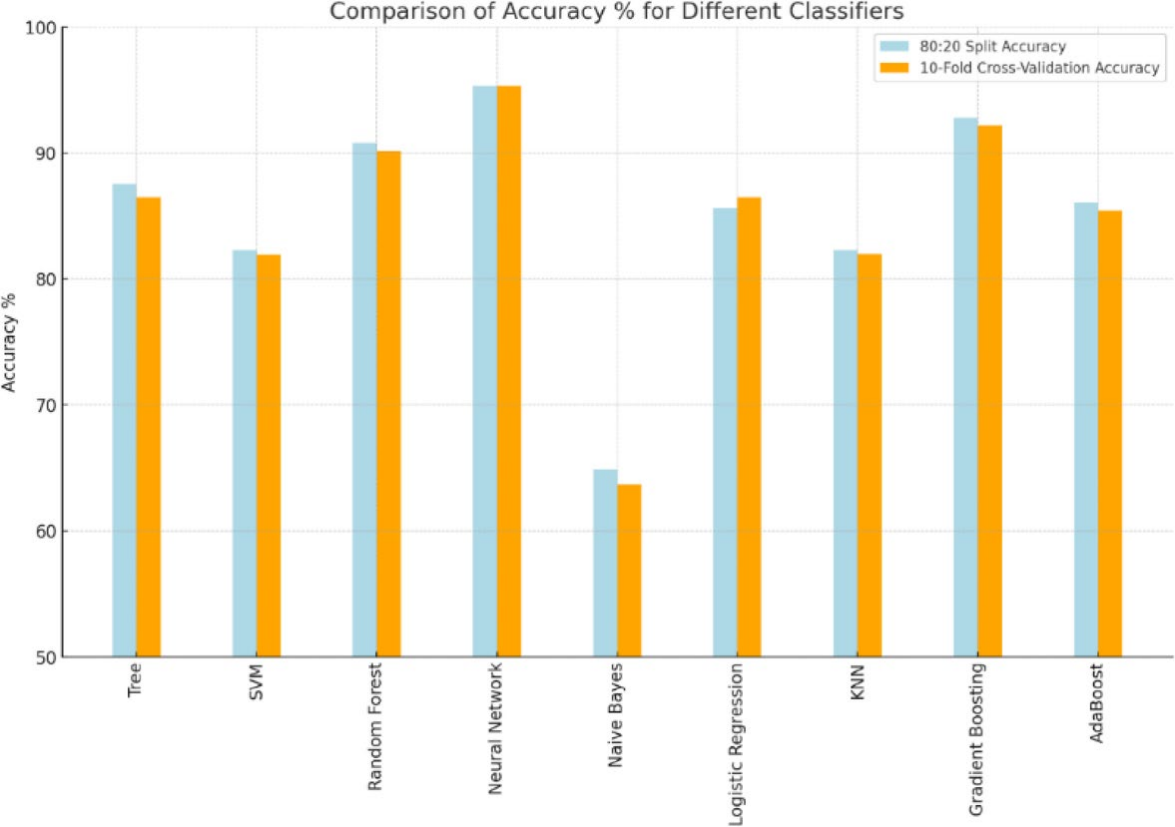
Differences in accuracy percentages using 80:20 split and cross validation.

Based on the results from 10-fold cross-validation, Neural Networks and Gradient Boosting emerge as the top-performing models, achieving the highest accuracy (95.3% and 92.2%, respectively) and consistently high F1-scores, precision, and recall across all classes. Random Forest also performs exceptionally well, with 90.2% accuracy and strong metrics overall. On the other hand, Naïve Bayes significantly underperforms, with the lowest accuracy (63.7%) and poor precision, recall, and F1-scores, making it less reliable for this specific dataset. SVM, KNN, Logistic Regression, and AdaBoost deliver moderate performance, with accuracy between 81.9% and 86.5%. While these models provide solid results, they are not as effective as Neural Networks or Gradient Boosting. In terms of consistency, most models show balanced performance across all classes, with minor variations in precision, recall, and F1-scores for specific classes (A, B, and C). However, SVM shows some struggle in Class A, while Naïve Bayes struggles across the board, particularly in Class B. For this churn prediction problem, Neural Networks, Gradient Boosting, and Random Forest are the top choices due to their high accuracy, precision, recall, and F1-scores. Naïve Bayes is the least suitable model, and the remaining models (SVM, KNN, Logistic Regression, and AdaBoost) offer reasonable performance but are not as strong as the top performers. The final model selection should consider both the task’s accuracy requirements and computational efficiency, with Neural Networks and Gradient Boosting being the most reliable for this problem. The differences in accuracy percentages using 80:20 split and 10-fold cross validation is shown in Fig. 10.

### Results based on another dataset

To assess the generalization capability of the proposed methodology, it has been applied to an additional dataset from INX Future Inc. This dataset comprises 1,200 employee performance records with 28 attributes, reflecting key employee characteristics relevant to churn prediction. The dataset is publicly available on Kaggle. The same methodology integrating feature selection using ChatGPT-4, criteria weighting and employee ranking using AHP-TOPSIS, and classification of the employees using various ML classifiers was tested on the new dataset to evaluate its scalability and adaptability.

**Fig. 11:**
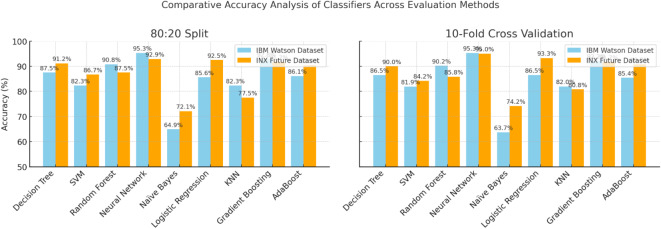
Comparative accuracy analysis of ML classifiers across different evaluation methods.

Table 6 presents the performance measures of the different ML classifiers when applied to the INX Future Inc. dataset after splitting the dataset into an 80:20 ratio and categorizing the employees into three distinct groups. Additionally, the evaluation metrics of the aforementioned ML models using 10-fold cross-validation are illustrated in Table 7. Figure 11 presents the accuracy comparison across the two datasets used in this study based on 80:20 split and 10-fold cross validation.

Based on the results from the 80:20 split of the second dataset, the ranking of top-performing classifiers differs from the first dataset, highlighting the dataset-dependent nature of classifier performance. Unlike the first dataset, where Neural Networks and Gradient Boosting were the dominant models, the second dataset sees Neural Networks (92.9%) and Logistic Regression (92.5%) achieving the highest accuracy. Additionally, Gradient Boosting (91.7%) and Decision Tree (91.2%) continue to perform well but do not outperform Logistic Regression as they did in the first dataset.

The same performance was obtained Based on the results from 10-fold cross-validation for the second dataset. Neural Networks remain the best model for both datasets. Conversely, Random Forest, which was a strong performer in the first dataset (90.2%), drops in ranking for the second dataset (85.8%), appears more dataset-dependent indicating that its effectiveness varies with different data distributions.

The shifts in classifier rankings between datasets highlight that model performance is influenced by dataset characteristics. While Neural Networks consistently excel, models like Logistic Regression, Random Forest, and Gradient Boosting fluctuate in ranking, reinforcing the need for statistical validation. These variations justify applying non-parametric tests like the Friedman test, which evaluates classifier performance across multiple datasets to determine whether differences in accuracy are significant or dataset-dependent. This ensures a more informed and reliable selection of the best-performing model.

**Table 6:** New dataset performance metrics (80:20 splits).

Classifier	Classes	Precision	Recall	F1-score	Accuracy	Accuracy %
Tree	All Classes	0.913	0.912	0.912	0.912	91.2
	Class A	0.932	0.917	0.924	0.912	
	Class B	0.902	0.925	0.914	0.912	
	Class C	0.914	0.883	0.898	0.912	
SVM	All Classes	0.876	0.867	0.865	0.867	86.7
	Class A	0.956	0.717	0.819	0.867	
	Class B	0.819	0.942	0.876	0.867	
	Class C	0.912	0.867	0.889	0.867	
Random Forest	All Classes	0.884	0.875	0.874	0.875	87.5
	Class A	0.957	0.75	0.841	0.875	
	Class B	0.826	0.95	0.884	0.875	
	Class C	0.927	0.85	0.887	0.875	
Neural Network	All Classes	0.931	0.929	0.929	0.929	92.9
	Class A	0.982	0.9	0.939	0.929	
	Class B	0.912	0.95	0.931	0.929	
	Class C	0.917	0.917	0.917	0.929	
Naïve Bayes	All Classes	0.720	0.721	0.719	0.721	72.1
	Class A	0.684	0.65	0.667	0.721	
	Class B	0.735	0.692	0.712	0.721	
	Class C	0.729	0.85	0.785	0.721	
Logistic Regression	All Classes	0.927	0.925	0.925	0.925	92.5
	Class A	0.981	0.867	0.92	0.925	
	Class B	0.911	0.942	0.926	0.925	
	Class C	0.905	0.95	0.927	0.925	
KNN	All Classes	0.794	0.775	0.772	0.775	77.5
	Class A	0.923	0.6	0.727	0.775	
	Class B	0.726	0.883	0.797	0.775	
	Class C	0.8	0.733	0.765	0.775	
Gradient Boosting	All Classes	0.922	0.917	0.917	0.917	91.7
	Class A	1	0.867	0.929	0.917	
	Class B	0.879	0.967	0.921	0.917	
	Class C	0.929	0.867	0.897	0.917	
AdaBoost	All Classes	0.904	0.904	0.904	0.904	90.4
	Class A	0.919	0.95	0.934	0.904	
	Class B	0.908	0.9	0.904	0.904	
	Class C	0.881	0.867	0.874	0.904	

**Table 7:** New dataset performance metrics (10-fold cross-validation).

Classifier	Classes	Precision	Recall	F1-score	Accuracy	Accuracy %
Tree	All Classes	0.905	0.9	0.9	0.9	90
	Class A	0.829	0.967	0.892	0.9	
	Class B	0.944	0.85	0.895	0.9	
	Class C	0.903	0.933	0.918	0.9	
SVM	All Classes	0.854	0.842	0.84	0.842	84.2
	Class A	0.794	0.9	0.844	0.842	
	Class B	0.918	0.75	0.826	0.842	
	Class C	0.784	0.967	0.866	0.842	
Random Forest	All Classes	0.861	0.858	0.859	0.858	85.8
	Class A	0.788	0.867	0.825	0.858	
	Class B	0.864	0.85	0.857	0.858	
	Class C	0.929	0.867	0.897	0.858	
Neural Network	All Classes	0.951	0.95	0.95	0.95	95
	Class A	0.906	0.967	0.935	0.95	
	Class B	0.966	0.933	0.949	0.95	
	Class C	0.967	0.967	0.967	0.95	
Naïve Bayes	All Classes	0.746	0.742	0.742	0.742	74.2
	Class A	0.676	0.767	0.719	0.742	
	Class B	0.778	0.7	0.737	0.742	
	Class C	0.75	0.8	0.774	0.742	
Logistic Regression	All Classes	0.938	0.933	0.933	0.933	93.3
	Class A	0.879	0.967	0.921	0.933	
	Class B	0.981	0.883	0.93	0.933	
	Class C	0.909	1	0.952	0.933	
KNN	All Classes	0.811	0.808	0.809	0.808	80.8
	Class A	0.767	0.767	0.767	0.808	
	Class B	0.794	0.833	0.813	0.808	
	Class C	0.889	0.8	0.842	0.808	
Gradient Boosting	All Classes	0.922	0.917	0.917	0.917	91.7
	Class A	0.853	0.967	0.906	0.917	
	Class B	0.917	0.917	0.917	0.917	
	Class C	1	0.867	0.929	0.917	
AdaBoost	All Classes	0.909	0.908	0.908	0.908	90.8
	Class A	0.931	0.9	0.915	0.908	
	Class B	0.902	0.917	0.909	0.908	
	Class C	0.9	0.9	0.9	0.908	

### Friedman test for classifier performance evaluation

To assess the statistical significance of the differences in classifier performance across multiple evaluation conditions, the Friedman test was conducted. This non-parametric statistical test evaluates whether significant differences exist among multiple classifiers over different experimental conditions^32^. It was applied to the accuracy results obtained from four evaluation scenarios: 80:20 split (Dataset 1), 10-fold cross-validation (Dataset 1), 80:20 split (Dataset 2), and 10-fold cross-validation (Dataset 2). The test yielded a Friedman statistic of 2.045 with a p-value of 0.563 (χ² = 2.045, *p* = 0.563), indicating that the observed differences in classifier performance across evaluation conditions are not statistically significant at the 0.05 level. This suggests that the choice of evaluation method (80:20 vs. 10-fold cross-validation) and dataset variation did not significantly impact the relative ranking of classifiers. Neural Networks (NN) remains the best-performing classifier in terms of raw accuracy, making it ideal for high-precision applications. Since no statistically significant difference exists, organizations can choose a model based on practical considerations such as training time, scalability, and business requirements to determine employee churn prediction.

### Time complexity

The proposed methodology follows a multi-stage process integrating Generative AI (ChatGPT-4), AHP, TOPSIS, and Machine Learning classifiers, each contributing to the overall computational complexity. Feature selection using GPT-4 operates with a complexity of O(n log n) due to ranking and sorting operations. AHP for criteria weighting is the most computationally expensive step, requiring O(n³) due to the construction of the pairwise comparison matrix, eigenvector computations, and consistency checking. TOPSIS, used for employee ranking, has a complexity of O(m × n) + O(m log m), where m is the number of employees and n is the number of features, making it more efficient compared to AHP. In the machine learning model training, Random Forest and Gradient Boosting have a training complexity of O(k × m log m), while Neural Networks, which achieve the highest predictive accuracy, require O(i × m × n²) due to iterative weight updates. The overall complexity of the methodology is O(n³) + O(m × n) + O(m log m) + O(i × m × n²), where AHP and Neural Network training’s contribute the highest computational burden. To improve efficiency, future research should focus on optimizing AHP’s weight computation and exploring faster ranking and deep learning techniques to reduce computational costs while maintaining accuracy. A summary of the overall computational complexity time is provided in Table 8.

**Table 8:** Overall computational complexity time.

Stage	Method Used	Time Complexity
Feature Selection	Generative AI (ChatGPT-4)	O(n log n)
Feature Weighting	AHP	O(n³)
Employee Ranking	TOPSIS	O(m × n) + O(m log m)
Machine Learning	Depends on model	O(i × m × n²) (NN), O(k × m log m) (RF, GB)
Overall Complexity	Depends on model	O(n³) + O(m × n) + O(m log m) + O(i × m × n²)

## Conclusion

By employing a combination of real and imitative experts, the methodology effectively captures diverse perspectives to evaluate and rank alternatives. This heterogeneity is reflected not only in the diversity of the data but also in the varied expertise of the real and imitative experts involved in the process. The methodology follows a structured approach, comprising four main stages: data collection, generative AI, MCGDM, and machine learning models for predicting employee churn. By utilizing a combination of real-world datasets and generative AI models representing experts from different fields, the methodology ensures that multiple viewpoints and criteria are considered in the decision-making process. GPT-4’s capabilities were used to generate a group of imitative experts with various areas of expertise, enriching the decision-making process.

The use of AHP and TOPSIS ensures that criteria weights and rankings are determined systematically, leading to the development of reliable predictive models for employee churn. AHP is employed to calculate the criteria weights, which are then used by TOPSIS to rank alternatives and classify employees into three categories based on their likelihood to leave work. The results from TOPSIS enable machine learning models to capture these categorization patterns, reducing the computational burden in future predictions by using trained ML models instead of recalculating with TOPSIS.

Neural Networks achieved the highest predictive accuracy for employee churn across both evaluated datasets, outperforming other machine learning classifiers. However, statistical analysis via the Friedman test confirmed no significant differences in performance among the models. Consequently, organizations may prioritize practical criteria including computational efficiency, scalability, and alignment with operational requirements when selecting an optimal model for employee churn prediction tasks.

Overall, the methodology, from data collection to final classification, provides a scalable and adaptable solution for organizations seeking to reduce employee turnover through data-driven decision-making. It stands out by integrating advanced AI techniques with traditional decision-making frameworks, enabling organizations to make informed and strategic decisions based on both expert knowledge and insightful data analysis. To summarize, the proposed methodology demonstrates practical applicability and offers a viable framework for integration within corporate environments. By employing this approach, organizations can systematically track employee turnover patterns and implement strategies to mitigate it effectively. In conclusion, employee churn prediction is utilized as a test case for solving MCGDM problems using the proposed methodology; however, this approach can be effectively extended to a wide range of other MCGDM applications.

To enhance the effectiveness and applicability of the proposed methodology, future research should focus on several key improvements. Conducting cross-industry validation using datasets from healthcare, finance, retail, and IT will improve the model’s adaptability beyond a single corporate environment; churn prediction and ensure its applicability in diverse workforce settings. To optimize the computational efficiency of AHP and TOPSIS, alternative MCDM techniques can be explored to enhance AHP’s time complexity and the multiple steps in TOPSIS. Additionally, validating AI-generated feature selection by comparing GPT-4-selected features with traditional methods such as regression will enhance the reliability and consistency of the feature selection process. Furthermore, integrating deep learning models and hybrid AI models to enhance the model’s ability to capture long-term behavioral trends in employee churn prediction, as traditional ML models may struggle with complex temporal patterns. By implementing these improvements, future research can significantly increase the scalability, adaptability, and predictive power of churn prediction models, making them more effective for real-world HR analytics applications.

## Data Availability

IBM Watson dataset published in 2017 from the Smarter Workforce Institute at IBM is available in: https://github.com/shailysaigal/Job-Satisfaction-on-IBM-Watson-dataset/blob/80ea0fc85152e1047163deaaac462e95b0da46c1/IBM_HR_Final_cleaned_Data.xlsm. INX Future Inc. dataset is publicly available at Kaggle in: https://www.kaggle.com/datasets/abhishekgupta0695/inx-future-inc-employee-performance. In addition, The generated datasets from all virtual experts utilized in the current study are available from the corresponding author on reasonable request. For further inquiries, please contact Hagar G. Abu-Faty at hagar.gamal@ai.menofia.edu.eg.
